# Supplementary material for: A secondary structure-based position-specific scoring matrix applied to the improvement in protein secondary structure prediction
Source: PLoS One. 2021 Jul 28;16(7):e0255076. doi: 10.1371/journal.pone.0255076 (PMC8318245; doi:10.1371/journal.pone.0255076)
Supplement: S1 Fig — (PDF) [file pone.0255076.s001.pdf]

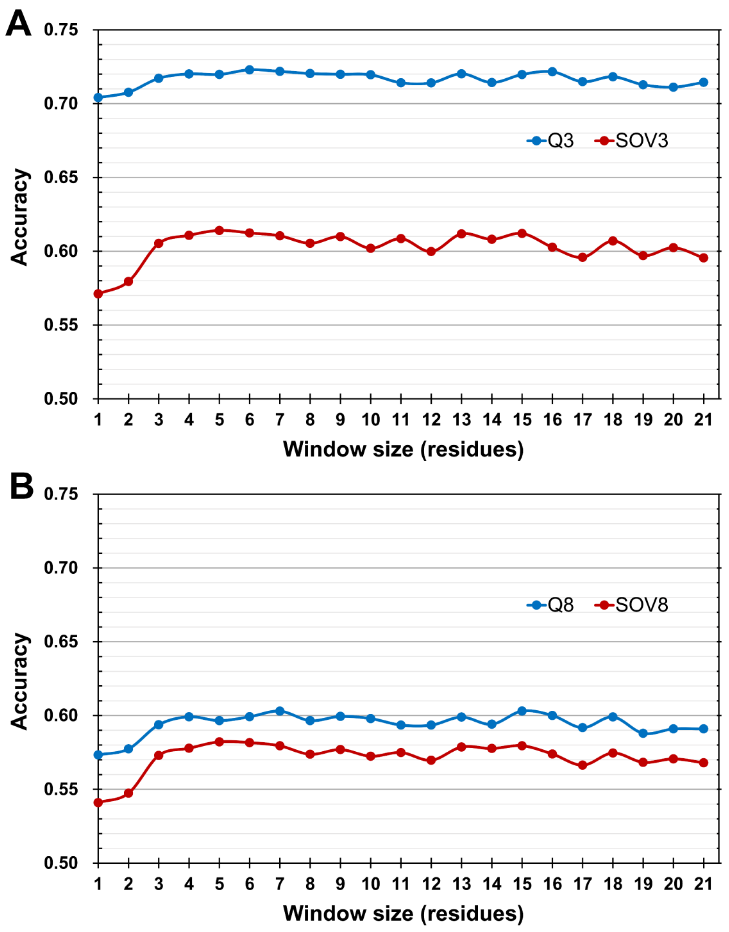

S1 Fig. Performance of different window sizes.

(A) Three-state accuracy. **(B)** Eight-state accuracy. The SSE-PSSM predictive model was preliminarily tested with window sizes from 1 to 21 residues to determine the optimal windows size for the SSE-PSSM feature set. In this test, the training query dataset QuerySet-T and TargetSet-nr25 were used, and the applied SSE alphabet was the SSE codes defined by DSSP [49]. Finally, a window of 5 residues was selected.
