# Supplementary material for: A secondary structure-based position-specific scoring matrix applied to the improvement in protein secondary structure prediction
Source: PLoS One. 2021 Jul 28;16(7):e0255076. doi: 10.1371/journal.pone.0255076 (PMC8318245; doi:10.1371/journal.pone.0255076)
Supplement: S4 File — (PDF) [file pone.0255076.s005.pdf]

BLOSUM25 of SSEs (BLOSUM25-SSE), v1.0, Apr. 2021:

	E	C	T	H	S	G	B	I
E	1.2	−0.9	−3.1	−6.8	−1.6	−3.5	−0.7	−2.5
C	−0.9	1.9	−1.1	−3.0	0.7	−1.5	0.5	0.1
T	−3.1	−1.1	2.5	−0.7	1.0	1.7	−0.3	2.1
H	−6.8	−3.0	−0.7	2.9	−2.1	−0.9	−3.6	1.5
S	−1.6	0.7	1.0	−2.1	2.9	−0.1	−0.2	1.0
G	−3.5	−1.5	1.7	−0.9	−0.1	3.5	−2.1	0.7
B	−0.7	0.5	−0.3	−3.6	−0.2	−2.1	5.5	0.2
I	−2.5	0.1	2.1	1.5	1.0	0.7	0.2	12.1

SSE Propensities:

E	0.392523
C	0.204186
T	0.127606
H	0.123848
S	0.079868
G	0.058207
B	0.013698
I	0.000063

This substitution matrix of secondary structural elements (SSE) was made based on a modified BLOSUM algorithm [88] stated in [77]. The main steps are described below,

1. Identify structural homologs from the nrPDB90-2015 dataset in an all-against-all manner using the SARST protein structural alignment search tool [77].

2. Calculate the sequence identity of every pair of homologs based on the SARST structure alignment and collect the pairs with sequence identity ≥25%.

3. Compute the propensity of each SSE in the collected homolog pairs. Let $p(i)$ denote the propensity of SSE i.

4. Compute the observed occurrence frequency of every aligned pair of SSEs based on the collected homolog pairs. Let $f_o(i, j)$ denote the observed frequency of SSE pair i and j.

5. The substitution score $S(i, j)$ of the SSE pair i and j is calculated as the logarithm of the ratio between their observed occurrence frequency and theoretical occurrence frequency according to the following BLOSUM equation,

$$S(i,j) = \log_2\left(\frac{f_o(i,j)}{p(i) \times p(j)}\right)$$

6. Following the nomenclature of BLOSUM matrices, the matrix of SSEs generated based on homologs of ≥25% sequence identity is named BLOSUM25-SSE.
