# Supplementary material for: A secondary structure-based position-specific scoring matrix applied to the improvement in protein secondary structure prediction
Source: PLoS One. 2021 Jul 28;16(7):e0255076. doi: 10.1371/journal.pone.0255076 (PMC8318245; doi:10.1371/journal.pone.0255076)
Supplement: S6 Table — (PDF) [file pone.0255076.s012.pdf]

S6 Table. Influence of the proportion of low or null occurrence codes in position propensity matrix on the SSP accuracy of several residue alphabets.

The datasets used to perform this experiment was the QuerySet-T and TargetSet-nr25. There are 720 proteins in the QuerySet-T. For each residue position of a protein in this query dataset, the proportions of low and null occurrence codes were computed. The proportion values listed in this table were the values averaged over all residues. A code in a given residue position is considered to be of low occurrence if its occurrence frequency is lower than $1/n_s$, where n_s is the number of code symbols in the alphabet.

Alphabet	Q3	SOV3	Number of codes	Proportion of low occurrence codes	Proportion of null occurrence codes
Eight-state SSE codes defined by DSSP [49]	0.793	0.748	8	0.705	0.458
Kappa-alpha codes defined by 3D-BLAST [76]	0.744	0.681	22	0.733	0.611
Ramachandron codes defined by SARST [77]	0.745	0.677	23	0.741	0.640
Amino acids	0.655	0.554	20	0.767	0.678
