# Supplementary material for: A secondary structure-based position-specific scoring matrix applied to the improvement in protein secondary structure prediction
Source: PLoS One. 2021 Jul 28;16(7):e0255076. doi: 10.1371/journal.pone.0255076 (PMC8318245; doi:10.1371/journal.pone.0255076)
Supplement: S9 Table — (PDF) [file pone.0255076.s015.pdf]

S9 Table. Accuracy of feature sets comprising different combinations of SSE-PSSMs.

In this experiment, several SSE-PSSM feature sets were generated base on different SSE alphabets, inclusive of the conventional three-state codes (SSE3), the eight-state codes (SSE8) defined by DSSP [49], the kappa-alpha codes (KA) by 3D-BLAST [76], and the Ramachandran codes defined by SARST [77]. Various combinations of these feature sets were evaluated by performing three-state predictions using QuerySet-T and TargetSet-nr25 (see **Materials and methods**). The **numbers in red** indicate the best performers in Q3 and SOV3, while the **numbers in blue** indicate the second best. It seemed that the combination of SSE8-, KA- and SARST-PSSM or the combination of SSE8- and KA-PSSM would be the best choice to develop an SSE-PSSM feature set for accurate SSP. However, the Q3 and SOV3 values of these combined feature sets were just slightly higher than those of the SSE8 feature set. Considering that among these combinations and the SSE8 alphabet, the latter is the easiest to implement and will achieve the least time cost in machine learning and prediction because it produces the smallest number of features, we decided to use the SSE8 alphabet to perform all experiments in this study.

Combination of SSE-PSSMs	Q3	SOV3
SSE3	0.790	0.741
SSE3, SSE8	0.790	0.735
SSE3, KA	0.792	0.742
SSE3, SARST	0.792	0.744
SSE3 ,SSE8, KA	0.791	0.733
SSE3, SSE8, SARST	0.791	0.735
SSE3, SSE8, KA, SARST	0.793	0.735
SSE8	0.793	**0.748**
SSE8, KA	**0.795**	**0.747**
SSE8, SARST	0.793	0.741
SSE8, KA, SARST	**0.796**	**0.747**
KA	0.734	0.657
SARST	0.737	0.647
KA, SARST	0.740	0.657
