# Supplementary material for: A secondary structure-based position-specific scoring matrix applied to the improvement in protein secondary structure prediction
Source: PLoS One. 2021 Jul 28;16(7):e0255076. doi: 10.1371/journal.pone.0255076 (PMC8318245; doi:10.1371/journal.pone.0255076)
Supplement: S10 Table — (PDF) [file pone.0255076.s016.pdf]

S10 Table. Accuracy of state-of-the-art SSP methods running in indirect combination with the SSE-PSSM features.

A. Results of the independent test based on QuerySet-I.

Method	Query dataset: QuerySet-I, Target dataset: TargetSet-nr25					
**Measure**	**Q3, native**	**Q3, ML**	**difference**	**SOV3, native**	**SOV3, ML**	**difference**
Scorpion	0.748	0.748	0.000	0.716	0.716	0.000
Spider2	0.755	0.749	–0.006	0.688	0.679	–0.009
SpineX	0.742	0.735	–0.007	0.692	0.695	0.003
PSIPRED	0.751	0.746	–0.005	0.694	0.702	0.008
DeepCNF	0.761	0.764	0.003	0.716	0.727	0.011
RaptorX	0.733	0.739	0.006	0.675	0.694	0.019
SSpro8	0.725	0.728	0.003	0.661	0.682	0.021
SSpro8T	0.910	0.910	0.000	0.875	0.876	0.001
MUFOLD-SS	0.775	0.766	–0.009	0.729	0.704	–0.025
NetSurfP2	0.787	0.786	–0.001	0.749	0.738	–0.011
Porter5	0.784	0.781	–0.003	0.751	0.743	–0.008
Spider3	0.785	0.776	–0.009	0.754	0.735	–0.019

The “native” values were obtained by directly using the SSP programs. The “ML” values were obtained by wrapping those methods with a second-level machine learning pipeline.

B. Results of training based on the training dataset QuerySet-T.

Method	Query dataset: QuerySet-T, Target dataset: TargetSet-nr25					
**Measure**	**Q3, native**	**Q3, ML**	**difference**	**SOV3, native**	**SOV3, ML**	**difference**
Scorpion	0.743	0.743	0.000	0.707	0.707	0.000
Spider2	0.750	0.744	–0.006	0.678	0.673	–0.005
SpineX	0.737	0.730	–0.007	0.683	0.685	0.002
Psipred	0.744	0.741	–0.003	0.686	0.692	0.006
DeepCNF	0.757	0.761	0.004	0.707	0.717	0.010
RaptorX	0.727	0.736	0.009	0.669	0.689	0.020
SSpro8	0.721	0.726	0.005	0.661	0.678	0.017
SSpro8T	0.915	0.916	0.001	0.883	0.885	0.002
MUFOLD-SS	0.771	0.763	–0.008	0.722	0.699	–0.023
NetSurfP2	0.780	0.786	0.006	0.737	0.734	–0.003
Porter5	0.783	0.781	–0.002	0.746	0.735	–0.011
Spider3	0.781	0.766	–0.015	0.745	0.727	–0.018
